# Persistence of Viral Reservoirs in Multiple Tissues after Antiretroviral Therapy Suppression in a Macaque RT-SHIV Model

**DOI:** 10.1371/journal.pone.0084275

**Published:** 2013-12-18

**Authors:** Christopher Kline, Jean Ndjomou, Tamera Franks, Rebecca Kiser, Vicky Coalter, Jeremy Smedley, Michael Piatak, John W. Mellors, Jeffrey D. Lifson, Zandrea Ambrose

**Affiliations:** 1 Division of Infectious Diseases, Department of Medicine, University of Pittsburgh School of Medicine, Pittsburgh, Pennsylvania, United States of America; 2 AIDS and Cancer Virus Program, Leidos Biomedical Research, Incorporated, (formerly SAIC-Frederick, Incorporated), Frederick National Laboratory for Cancer Research, Frederick, Maryland, United States of America; 3 Laboratory Animal Sciences Program, Leidos Biomedical Research, Incorporated, (formerly SAIC-Frederick, Incorporated), Frederick National Laboratory for Cancer Research, Frederick, Maryland, United States of America; Uniformed Services University, United States of America

## Abstract

Although antiretroviral therapy (ART) can suppress HIV-1 replication sufficiently to eliminate measurable plasma viremia, infected cells remain and ensure viral recrudescence after discontinuation of ART. We used a macaque model of HIV-1/AIDS to evaluate the location of infected cells during ART. Twelve macaques were infected with RT-SHIV_mne_, a SIV containing HIV-1 reverse transcriptase, conferring sensitivity to non-nucleoside reverse transcriptase inhibitors (NNRTIs). Ten to fourteen weeks post-infection, 6 animals were treated with 3 or 4 antiretroviral drugs for 17-20 weeks; 6 control animals remained untreated. Viral DNA (vDNA) and RNA (vRNA) were measured in peripheral blood mononuclear cells (PBMC) and at necropsy in multiple tissues by quantitative PCR and RT-PCR. The majority of virally infected cells were located in lymphoid tissues with variable levels in the gastrointestinal tract of both treated and untreated animals. Tissue viral DNA levels correlated with week 1 plasma viremia, suggesting that tissues that harbor proviral DNA are established within the first week of infection. PBMC vDNA levels did not correlate with plasma viremia or tissue levels of vDNA. vRNA levels were high in lymphoid and gastrointestinal tissues of the untreated animals; animals on ART had little vRNA expressed in tissues and virus could not be cultured from lymph node resting CD4+ cells after 17-20 weeks on ART, indicating little or no ongoing viral replication. Strategies for eradication of HIV-1 will need to target residual virus in ART suppressed individuals, which may not be accurately reflected by frequencies of infected cells in blood.

## Introduction

Current treatment for HIV infection is not curative. Although plasma viremia can be suppressed to very low or undetectable levels in HIV-infected individuals by effective antiretroviral therapy (ART), infected cells remain in the body and treatment discontinuation is almost always associated with viral recrudescence and the risk of disease progression [1-5]. Cells and tissues that harbor proviral HIV-1 DNA during suppressive ART capable of reinitiating productive systemic infection when treatment is stopped, are considered viral reservoirs and have been incompletely characterized, due in part to the difficulty in obtaining sufficient amounts of the relevant tissues and cells for comprehensive testing. It has been shown that early treatment of HIV-1 infection can reduce the size of viral reservoirs [6-9], suggesting that they begin to be established very early in infection, although they may continue to develop thereafter. Evidence has also been provided in support of the competing but not mutually exclusive ideas that these reservoirs are maintained by ongoing viral replication during ART [10-14] or by expression of virus from long-lived cells infected prior to initiation of ART [15-19].

Animal models enable more extensive tissue sampling, including tissue collection at scheduled necropsy, than is typically feasible in a clinical setting and offer promise for facilitating studies of viral reservoirs and evaluation of viral eradication strategies. Macaques are widely used in nonhuman primate models for AIDS after infection with simian immunodeficiency viruses (SIV) or chimeric SIV containing HIV-1 sequences (simian-human immunodeficiency viruses or SHIV). SIV or SHIV infection of macaques recapitulates key aspects of human HIV-1 infection including progressive disease with clinically significant immunodeficiency and death from opportunistic infections or neoplasms despite the development of antiretroviral immune responses, such as neutralizing antibodies and cytotoxic T lymphocytes against the virus. While SIV shares a high degree of structural and sequence identity to HIV-1, the differences are significant enough to limit the use of some therapies in SIV-infected macaque models. For example, non-nucleoside reverse transcriptase inhibitors (NNRTIs) are only active against reverse transcriptases (RT) from HIV-1 and not those from HIV-2 or SIV [20]. To overcome this limitation, the RT coding region of different SIV clones has been replaced with that of HIV-1 to produce RT-SHIVs, which can be targeted by RT inhibitors, including NNRTIs, for use in macaque studies [21,22].

For meaningful studies of persistent viral reservoirs in nonhuman primate models, it is necessary to achieve and maintain clinically relevant levels of viral suppression. Recent reports interpret lack of evidence for viral evolution to suggest that ongoing viral replication does not occur during effectively suppressive ART in humans [15,16,18,19]. Similar findings were obtained in RT-SHIV-infected monkeys with sustained suppression of plasma viremia [23]. However, continued virus replication in the presence of incomplete suppression can lead to infection of new target cells and re-seeding of reservoirs, confounding efforts to identify reservoirs that were established prior to initiation of ART. 

In this study, we quantified the viral DNA (vDNA) in tissues from RT-SHIV-infected pigtailed macaques in which viral replication was suppressed with the clinically used triple combination therapy of tenofovir (TFV), emtracitabine (FTC), and efavirenz (EFV), with or without intensification with an integrase inhibitor, to characterize reservoirs of infected cells (vDNA+) persisting in the face of suppressive ART. In addition, we measured viral RNA (vRNA) levels in these compartments. Finally, we evaluated potential correlations between levels of virally infected cells in tissues and peripheral blood mononuclear cells (PBMC) with the level of plasma viremia. 

## Materials and Methods

### Virus

RT-SHIV_mne_ is a chimeric virus in which the RT coding region of SIV_mne027_ was replaced with that of HIV-1_HxB2_ [21,24]. The challenge stock was produced by transfection of a full-length proviral plasmid into 293T cells, expanded in CEMx174 cells, and titered in TZM-bl cells.

### Animals

Twelve male pigtailed macaques (*Macaca nemestrina*) between 3-9 years old and weighing between 5 - 14 kg were infected intravenously with approximately 1×10^5^ infectious units of RT-SHIV_mne_. Males were studied due to limited availability of female pigtailed macaques and because having a mixture of genders would reduce the power of findings from genital tract tissues. All animals were housed at the National Institutes of Health (NIH) in accordance with the American Association of Accreditation of Laboratory Animal Care standards and all procedures were performed according to protocols approved by the Institutional Animal Care and Use Committee of the National Cancer Institute (Assurance #A4149-01). The animals were negative at study initiation for serum antibodies to HIV type 2, SIV, type D retrovirus, and simian T-lymphotropic virus type 1. Animals were housed in an AAALAC accredited facility and in compliance with the guidelines in the Guide for the Care and Use of Laboratory Animals. Animals were maintained in Animal Biosafety Level 2 housing according to the provisions of the 5th edition of the Biosafety in Microbiological and Biomedical Laboratories with a 12:12-hour light:dark cycle, relative humidity 30 - 70%, and a temperature of 23 - 26°C. Filtered drinking water was available ad libitum, and a standard commercially formulated nonhuman primate diet (Purina Labdiet 5045 “High Protein Monkey diet”, PMI Nutrition International, St. Louis, MO) was provided thrice daily and supplemented 3-5 times weekly with fresh fruit and/or forage material as part of the environmental enrichment program. Each cage (Allentown, Inc., Allentown, NJ) contained a perch, a two portable enrichment toys, one hanging toy, and a rotation of additional items (including stainless steel rattles, mirrors, and challenger balls). Additionally the animals were able to listen to radios during the light phase of their day and were provided with the opportunity to watch full-length movies at least three times weekly. Pain and distress were relieved by appropriate measures. All procedures were conducted while the animals were sedated with intramuscular injection of Telazol (tiletamine/zolazepam, 3-6mg/kg). Animals that were diagnosed by the veterinarian to be experiencing more than momentary pain and distress were evaluated and treated with appropriate analgesic drugs as indicated. The end point of the study was euthanasia at the protocol-specified time point by intravenous pentobarbital (80 mg/kg) in the saphenous vein after sedation with 3 mg/kg Telazol. 

Six animals were left untreated for 30-32 weeks post-infection. The remaining 6 animals received ART: 2 were treated daily with TFV, FTC, and EFV for 20 weeks, beginning at week 10 post-infection; 4 animals were treated daily with TFV, FTC, EFV, and the integrase inhibitor L-870812, for 17-18 weeks, beginning at week 13 or 14 post-infection. Treatments were continued until necropsy and administered as previously described: 20mg/kg of TFV and 40mg/kg of FTC administered once per day subcutaneously, 400mg of EFV administered one per day orally, and 100mg of L-870812 administered twice per day orally [24-27]. Orally administered drugs were given in treats and animals were observed to ensure that they consumed them.

Blood was drawn weekly or biweekly, from which plasma and PBMC were separated, aliquotted, and stored at -80° C or in liquid nitrogen, respectively. At week 30-32 post-infection, animals were euthanized and necropsies were performed, without prior perfusion, in which 3 equivalent parallel specimens were randomly selected from each of multiple tissues and processed as follows: 1) formalin fixed for histology, 2) flash frozen dry in liquid nitrogen for DNA isolation, and 3) flash frozen with RNAlater (Ambion, Austin, TX) in liquid nitrogen for RNA isolation. Tissues sampled included the gastrointestinal tract (duodenum, jejunum, ileum, colon, cecum/rectum), liver, lymphoid tissues (thymus, bone marrow, spleen, axillary, bronchial, inguinal, mandibular, mediastinal, colonic and mesenteric LN), brain (cerebrum, cerebellum, and midbrain), genital tract (testicle and seminal vesicles), and lung. PBMC were also isolated and frozen as cell pellets or viably cryopreserved in cell culture medium containing 10% DMSO.

### Plasma viral loads and T cell subset counts

Virus was pelleted from EDTA-anticoagulated plasma taken at each time point for each animal and quantitative RT-PCR (qRT-PCR) was performed to determine the number SIV RNA (*gag*) copy equivalents per ml (copy Eq/ml) of plasma essentially as previously described [28]. The assay limit for quantitation was 30 vRNA (*gag*) copy Eq/ml of plasma. 

T lymphocyte subsets (CD3, CD4, CD8, CCR5 expressing T cells) were measured by a whole blood immunostaining procedure and flow cytometry analysis. Briefly, tubes were prepared with 10microliters each of the following antibody panels (BD Biosciences, San Jose, CA): Panel 1 anti-CD3 (Alexa Fluor 488 conjugate, Clone SP342; BD Biosciences, San Jose, CA)/anti-IgG2a control (PE conjugate, Clone X39)/anti-CD8 (PerCPCy55 conjugate, Clone SK1)/anti-CD4 (APC conjugate, Clone L200), and Panel 2 anti-CD3 (Alexa Fluor 488 conjugate, Clone SP342)/anti-CD195 (CCR5; PE conjugate, Clone 3A9)/anti-CD8 (PerCPCy55 conjugate, Clone SK1)/anti-CD4 (APC conjugate, Clone L200). 100 microliters of whole blood was then added, mixed, and incubated for 30 min at ambient temperature, followed by addition of 2 ml of FACS Lysing Solution (BD Biosciences) to lyse red blood cells. After incubation, cells were pelleted, washed with PBS containing 1% bovine serum albumin fraction IV (Sigma-Aldrich, St. Louise, MO) and sodium azide, then resuspended in 2% paraformaldehyde for fixation prior to analysis. To calculate absolute numbers of different T lymphocyte subsets, the percentages of each population were multiplied by the absolute lymphocyte count determined by a total white blood cell count with differential performed on the same specimen. 

### Isolation of replication-competent virus from resting CD4+ T cells from LN biopsies

Superficial (axillary or inguinal) LN were obtained by biopsy at weeks 12 or 13, 16 or 17 post-infection, or at necropsy (weeks 30-32 post-infection) and single cell suspensions were prepared using sterile mesh screens. Resting CD4+ T cells were isolated to > 95% purity using an immunomagnetic microbead procedure for depletion of non-CD4+ cells with antibodies against macaque CD8, CD11b, CD16, CD20, CD56, CD66abce, and HLA-DR (Miltenyi, Auburn, CA). The cells were suspended in T cell conditioned medium [29] and a limiting dilution co-culture assay with CEMx174 cells was performed as previously described [27]. Infectious units per million cells (IUPM) were determined by maximum likelihood analysis based on measurement of supernatant p27 CA levels by ELISA (Zeptometrix, Buffalo, NY). The assay had a limit of quantitation of 0.5 IUPM based on maximum likelihood analysis of a limiting dilution between 1×10^6^ cells - 3.2×10^2^/well in duplicate [29].

### Viral and host cell DNA isolation and measurement in PBMC and tissues

Total DNA (viral and genomic) was extracted from tissues with lysis buffer (Nuclei lysis solution, Promega, Madison, WI) after extensive washing using a TissueLyser (Qiagen, Valencia, CA). Tissue DNA was extracted using the Wizard genomic purification kit (Promega) and PBMC DNA was extracted using the Blood DNA kit (Qiagen) in a total of 100 microliters of supplied buffer. Recovery of macaque *CCR5* and viral gag DNA from PBMC, as described below, were similar for both extraction methods (data not shown).

qPCR assays were performed in duplicate on 5 microliters of each extracted DNA sample to quantify macaque *CCR5* DNA, viral gag DNA, and viral 2-LTR circles, without prior PCR pre-amplification. These assays were performed using qPCR mastermix (Bio-Rad, Hercules, CA) and primers and probes specific for each gene (Table S1 in File S1) on a CFX96 real-time PCR machine (Bio-Rad), using the following conditions: 95° C for 2 minutes followed by 44 cycles of 95° C for 15 seconds and 60° degrees for 1 minute. The limit of quantitation for each assay was 10 copies/sample for *CCR5* and 1 copy/sample for *gag* and 2-LTR circles, as determined by a serial endpoint dilution of standards in replicates of ten, of which approximately 60% were positive at a nominal template input of 1 copy/reaction (Table S2 in File S1). A plasmid encoding the macaque *CCR5* gene or PCR products for *gag* and 2-LTR circle junctions were used to make standard dilutions that were run in duplicate for each qPCR. Only data generated by assay runs with standard curves having R^2^ values of ≥ 0.995 were used (Figures S1 and S2 in File S1). 

### Viral and host RNA isolation and measurement in tissues

Viral and host cell RNA were extracted from tissues stored in RNAlater. Tissues were rinsed with nuclease-free water and homogenized using a TissueLyser in the presence of lysis buffer (RTL, Qiagen) and 20U of RNase inhibitor (Ambion). RNA was extracted from lymphoid tissues, lung, liver, and genital tract tissues using the RNeasy kit (Qiagen) in a total of 50 microliters RNase-free water. As this method did not yield good RNA recovery from gastrointestinal tract and brain tissues, TRI Reagent (Sigma-Aldrich, St. Louis, MO) was used to extract RNA from brain and gut tissues, as previously described [30]. Purified RNA was treated with 10U RNase-free DNase I (Roche, Indianapolis, IN) and then precipitated and washed again using the RNeasy kit. RNA extraction of each half of a lymph node from an infected animal with both extraction methods showed similar recoveries of host and viral RNA (data not shown).

qRT-PCR assays were performed in duplicate on each sample to quantify macaque *CD4* and *IPO-8* RNA and viral gag RNA. cDNA was synthesized using 3 microliters of the RNA samples as well as RNA transcript standards using the SuperScript III First Strand Synthesis kit (Invitrogen, Carslbad, CA) and reverse CD4, IPO-8, or gag primers (Table S1 in File S1) in a total of 12 microliters. qPCR was performed with 5 microliters of cDNA, using qPCR mastermix (SABiosciences, Frederick, MD) and primers and probes specific for each gene (Table S1 in File S1) on a CFX96 real-time PCR machine, using the following conditions: 95° C for 2 minutes followed by 44 cycles of 95° C for 15 seconds and 60° degrees for 1 minute. The limit of detection for the gag assay was approximately 1 copy/sample (Table S2 in File S1). *CD4* and *gag* transcripts for standard dilutions were synthesized from plasmids encoding the pigtailed macaque *CD4* or RT-SHIV_mne_ *gag* gene using the RiboMAX large scale RNA production system (Promega). The standards were run in duplicate and only data generated with standard curves having R^2^ values of ≥ 0.995 were used (Figure S3 in File S1).

### Statistics

To test the hypothesis of independence between tissue types and DNA or RNA levels between treated and untreated tissue types, we conducted a chi-square test. Non-normalized DNA and RNA measurements were analyzed. Non-numeric values were treated as either zero (below detection) or missing (not available or not determined). To compare the treated and untreated group, we conducted multiple unequal variance t tests to test whether there is a difference between two groups in terms of vDNA and vRNA counts. We adjusted the multiple tests by using the Hochberg and Benjamini method [31]. 

We used Pearson’s product moment correlation to study the strength of a linear association between the lymphoid tissue vDNA levels and plasma viremia at week 1, between the lymphoid tissue vDNA levels at necropsy and the area under the curve (AUC) for plasma vRNA over 32 weeks. The correlations were studied for both treated and untreated animals. Spearman’s rank correlation was also conducted as a check for the robustness of the product moment correlation due to violations of the assumption of normality.

## Results

### Infection and treatment of animals

**Figure 1:**
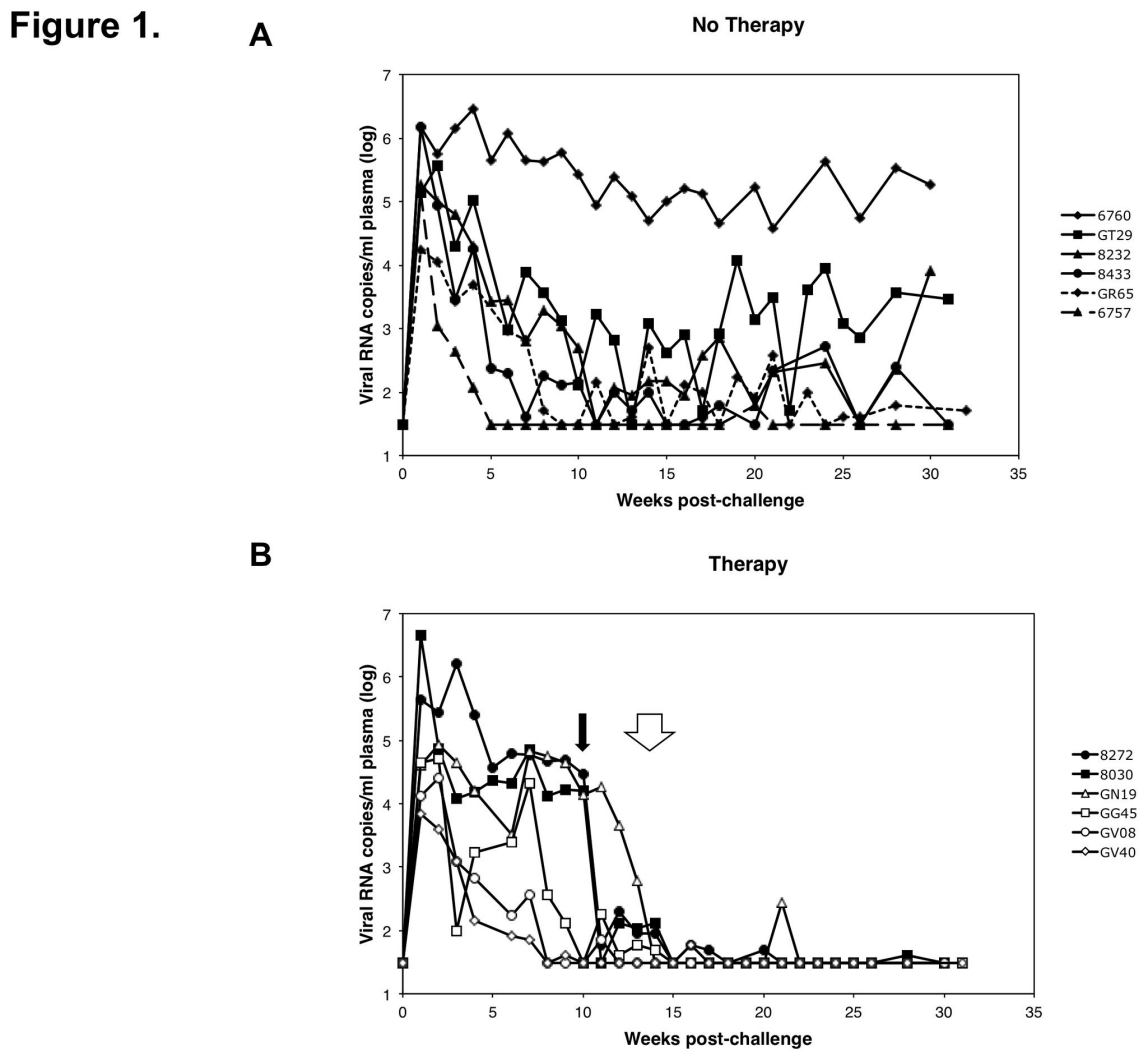
Plasma viremia was measured in all twelve macaques by qRT-PCR of RT-SHIV *gag* RNA. Animals were infected at week 0 and were (A) untreated or (B) treated with 3 or 4 antiretroviral drugs. Animals treated with 3 drugs (TFV, FTC, EFV) are denoted by closed symbols and treatment was initiated at week 10, denoted by the solid arrow. The animals treated with 4 drugs (TFV, FTC, EFV, and L-870812) are denoted by open symbols and treatment was initiated at week 13 (GV08 and GN19) or week 14 (GG45 and GV40), denoted by the open arrow. Treatment was continued daily until necropsy (week 30 or 31). The limit of detection of the assay was 30 vRNA copies/ml plasma.

Following intravenous infection, (week 0) plasma viremia was measured weekly or biweekly throughout the study (Figure 1). As observed in our previous studies [23,24], plasma RT-SHIV RNA levels were variable among animals. Higher levels of RT-SHIV viremia can result in significant pathogenesis and AIDS-like disease in pigtailed macaques, including gastrointestinal pathology [24]. The range in peak and post-acute levels of plasma viremia made it possible to evaluate potential correlations between these levels of plasma viremia and vRNA and vDNA levels in tissues taken at necropsy.

All animals became infected with variable peak and set point plasma viremia levels. One untreated animal (6757) had sustained undetectable plasma viremia by 5 weeks post-infection, while all others had consistently detectable levels of viremia during the study period. Treatment of 2 animals (8272 and 8030) with TFV, FTC, and EFV was initiated at week 10 and continued daily for 20-22 weeks until euthanasia and necropsy at 30-32 weeks post-infection. After 5-7 weeks of ART, plasma viremia was < 50 vRNA copies/ml. Because 3 drugs did not completely suppress high plasma viremia levels, treatment of 4 animals with TFV, FTC, EFV, and the integrase inhibitor L-870812 was initiated at week 13 (GV08 and GN19) or week 14 (GG45 and GV40) until euthanasia and necropsy after 17-18 weeks on ART (30-32 weeks post-infection). Both ART regimens led to initial decreases in plasma viremia to < 30 copy Eq/mL and plasma viremia remained < 30 copy Eq/mL for the majority of samples over the duration of follow up to necropsy for all animals. Animals GV08 and GV40 had undetectable plasma viremia when ART was initiated. Animal GN19 showed a viremic time point at 8 weeks after ART initiation (280 viral RNA copies/ml). All treated animals had < 30 copy Eq/mL of plasma vRNA on the day of necropsy. 

**Figure 2:**
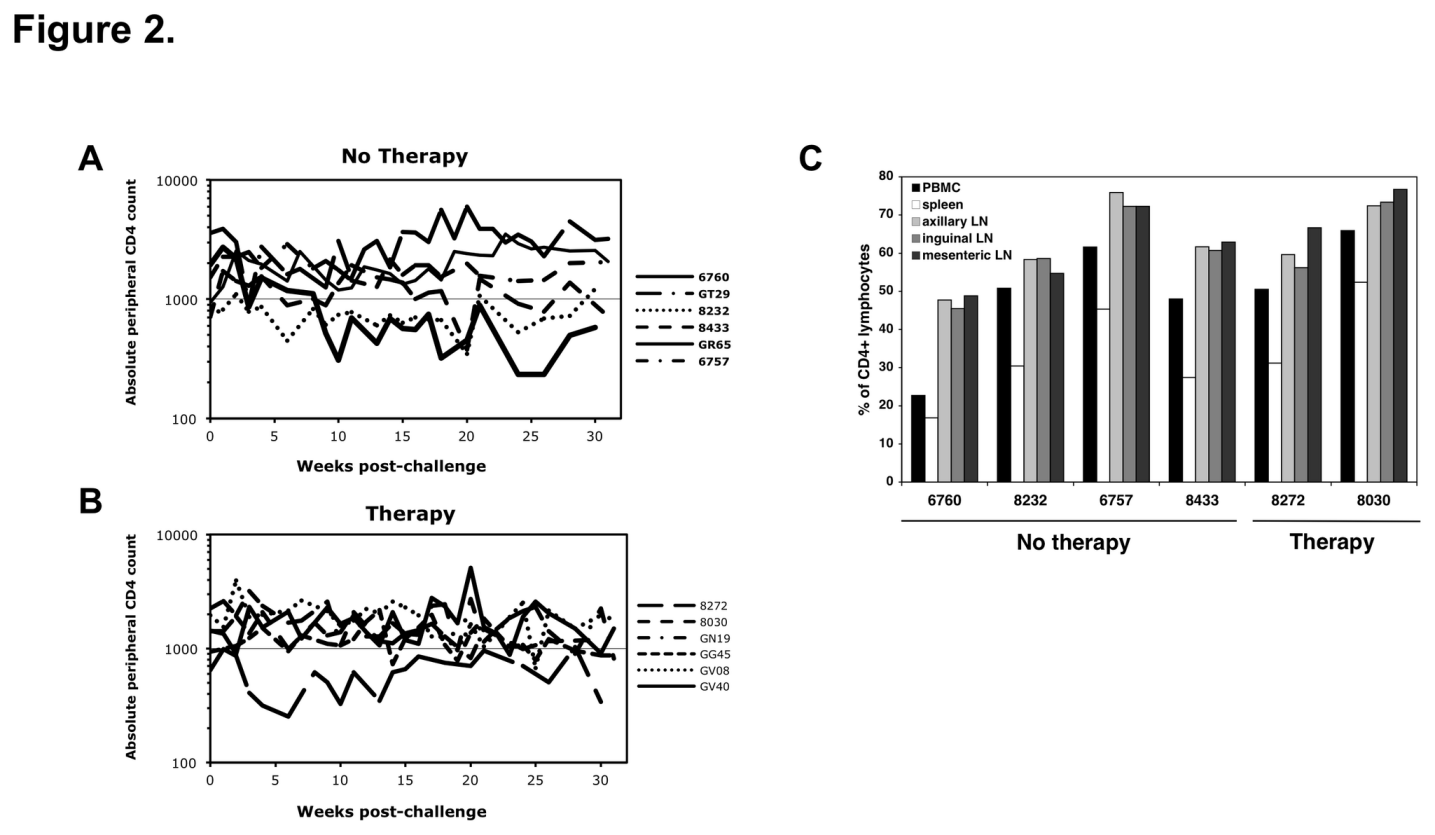
CD4 numbers in PBMC and tissues. Absolute CD4 counts in the blood of (A) untreated and (B) treated animals were plotted from multiple time points. (C) The percentage of CD4+ lymphocytes in PBMC, spleen and multiple lymph nodes taken at necropsy in 6 animals are shown.

Peripheral blood CD4+ T cells were monitored weekly or biweekly during the study for the untreated and treated animals (Figure 2A and B, respectively). The majority of the animals had relatively stable CD4+ T cell counts. Three of the untreated animals (6760, 8232, and 8433) and one of the treated animals (8272, 3 drugs) had at least one time point with <350 CD4+ T cells/mm^3^. Animal 6760 had less than 250 CD4+ T cells/mm^3^ at two time points (weeks 24 and 26), but they rebounded before necropsy. No counts less than 200 cells/mm^3^ were measured for any of the animals over the period of follow up. 

### Viral DNA is detected in many tissues and PBMC of untreated and ART-treated macaques

To determine the frequency and location of infected (vDNA+) cells remaining during ART that could contribute to viral reservoirs and provide a potential source of recrudescent virus if ART was discontinued, we isolated DNA from necropsy tissues (Table 1). RT-SHIV DNA was measured in each sample, using a qPCR assay targeting *gag*. To normalize for differences in the amount of tissue and/or amount of DNA extracted from the samples, a target sequence from macaque CCR5 gene, not known to be duplicated in the genome, was also measured in each sample by qPCR. Most tissue samples yielded greater than 10^6^ *CCR5* copies and data were only included from tissues in which > 10^5^ *CCR5* copies could be detected, without significant discordance between duplicates (Figure S1 in File S1). Data were normalized based on CCR5 measurements, assuming two copies of CCR5 target sequence per diploid cell. 

**Table 1:** *gag* DNA copies per 1x10^6^ *CCR5* DNA copies per tissue at necropsy (week 30/31).

	**Untreated**							**Treated**					
								**3 Drugs**		**4 Drugs**			
	**6760**	**8433**	**8232**	**6757**	**GT29**	**GR65**		**8272**	**8030**	**GN19**	**GG45**	**GV08**	**GV40**
Plasma RNA	**180000**	<30	**8200**	<30	**2900**	**50**		<30	<30	<30	<30	<30	<30
PBMC	**271**	**454**	**531**	**73**	**572**	**226**		**288**	**52**	**315**	**485**	**39**	**611**
Duodenum	**64**	-	**72**	**2**	-	-		**31**	-	nd	nd	**5**	-
Jejunum	**36**	-	**9**	-	-	nd		**14**	**19**	**589**	-	-	-
Ileum	**330**	-	**205**	**2**	**145**	-		**32**	-	**4559**	nd	**8**	-
Colon	**203**	-	**6**	**1**	**16**	**2**		**60**	-	**5615**	-	**3**	**1**
Cecum/Rectum	**103**	**31**	**24**	**5**	**52**	**10**		**9**	**17**	**64**	-	**1**	**2**
Liver	**8**	**12**	**2**	-	**1**	**1**		**5**	**2**	**8**	**2**	-	-
Lung	**9**	**32**	**38**	-	**4**	**2**		**9**	**25**	**42**	**1**	**1**	-
Thymus	**7**	nd	nd	nd	-	-		nd	**200**	nd	-	-	-
Bone marrow	**25**	**25**	**2**	**1**	-	**1**		**4**	**2**	**1**	**1**	-	-
Spleen	**275**	**198**	**64**	**11**	-	-		**52**	**48**	**79825**	-	-	**9**
Axillary LN	**833**	**374**	**86**	**15**	nd	**5**		**171**	**32**	**3333**	**13**	**2**	**5**
Bronchial LN	**1002**	**893**	**97**	**13**	-	-		**524**	**219**	**6149**	-	**12**	**5**
Inguinal LN	**611**	**818**	**21**	**14**	**40**	**9**		**183**	**46**	nd	**21**	nd	**6**
Mandibular LN	**884**	**605**	**115**	**9**	**17**	**41**		nd	**13**	**377**	**41**	**8**	**25**
Mediastinal/Colon LN	**48**	**760**	nd	-	-	**18**		nd	**50**	**9282**	**74**	**76**	**30**
Mesenteric LN	**538**	**437**	**251**	**9**	**4**	-		**629**	**29**	**12282**	**177**	**14**	**19**
Cerebellum	-	-	-	-	-	-		-	-	-	**48**	-	-
Midbrain	-	-	**4**	**6**	**10**	-		**-**	**13**	-	**6**	-	**1**
Cerebrum	**1**	-	-	**14**	-	-		**21**	**2**	-	-	**2**	**2**
Testicle	**1**	-	-	-	-	-		-	-	-	-	-	-
Seminal vesicle	-	**1**	**1**	-	-	-		**2**	**4**	nd	-	-	-
Kidney	**42**	**2**	**7**	-	nd	nd		**3**	**2**	nd	nd	nd	nd

nd, not determined due to insufficient genomic DNA recovery or lack of tissue

-, less than 1

All animals, regardless of treatment or plasma viral load, had detectable vDNA in multiple tissues, most prominently in the gastrointestinal (GI) tract, lung, and all lymphoid tissues of the animals (Table 1). Much lower levels of vDNA (1 to 25 copies/10^6^ diploid cell equivalents) were detected in bone marrow samples of 9/12 animals, compared to the spleen of 9/12 animals (9 to 79,800 copies/10^6^ cells) or various LN of all of the animals (2 to 12,300 copies/10^6^ cells). In non-lymphoid and non-mucosal tissues, such as the brain, testes and seminal vesicles, fewer vDNA copies were detected (1 to 48 copies/10^6^ cells or 1 to 4 copies/10^6^ cells, respectively) and often there was no detectable vDNA (23/36 brain samples and 19/23 testes and seminal vesicle samples). As we did not study CD4+ cells isolated from the tissues, the observed differences in the number of vDNA copies between tissues could be influenced by the relative frequency of CD4+ target cells present in different tissue specimens, such as different portions of the GI tract. 

The *gag* primers recognize both unintegrated vDNA, including 2-LTR circles, and proviral DNA. To determine what proportion of the measured viral DNA was due to 2-LTR circles, we measured 2-LTR circles by qPCR in all samples that had > 100 gag DNA copies per 10^6^ CCR5 copies using primers that span the 2-LTR circle junction, a target template that is not present in linear forms of the viral DNA genome [32]. The results from multiple tissues of animal 6760 are shown (Figure S2 in File S1). While 100-10,000 copies of *gag* are detected in these samples, a range of less than 1 and up to 304 copies of 2-LTR circles were detected. These accounted for 0.08 - 3.8% of the *gag* copies detected. Similar results were observed in samples from the other animals (data not shown). Thus, less than 4% of vDNA consists of 2-LTR circles and tissues containing less than 100 copies of total vDNA are likely to have negligible or undetectable levels of 2-LTR circles. The sample size for the groups treated with or without ART containing an integrase inhibitor did not provide sufficient power to compare the levels of 2-LTR circles between the two treatment regimens.

### Levels of vDNA in lymphoid tissues correlated with week 1 plasma viremia in untreated animals

**Figure 3:**
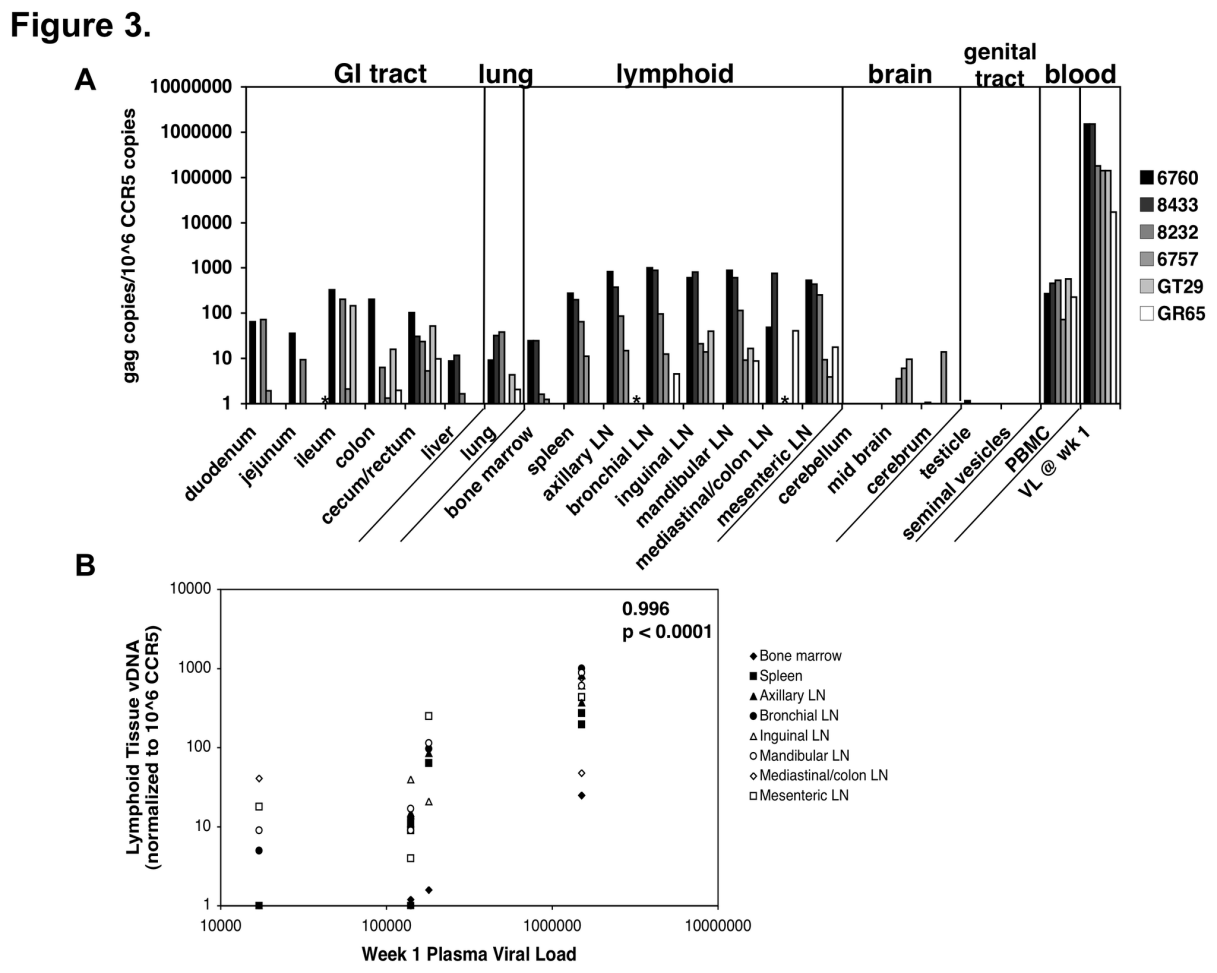
Lymphoid tissue viral DNA at necropsy is correlated with week 1 plasma viremia levels. (A) The ratio of *gag* copies per 10^6^ CCR5 copies for each tissue of the untreated RT-SHIV-infected macaques. The average of each qPCR reaction was used for the graph. In addition, the week 1 plasma viral load was included for each animal. Asterisks (*) denote samples that were not collected or in which no significant CCR5 DNA were measured. (B) The amount of *gag* vDNA detected in each of the lymphoid tissues for each animal was plotted against the week 1 plasma viremia level. Statistics determined a Spearman rank-order correlation of 0.996 with p value of < 0.0001.

Comparing week 1 plasma viremia with vDNA levels measured at necropsy in lymphoid tissues of the untreated animals, we noted an apparent correlation (Figure 3A). Spearman rank-order correlation analysis demonstrated a strong positive correlation between the lymphoid tissue vDNA levels and plasma viremia at week 1 (0.996 with p value < 0.0001) (Figure 3B). Analysis comparing lymphoid tissue vDNA levels at necropsy and area under the curve (AUC) of the plasma viremia levels over the entire 32 week period, a parameter reflecting cumulative plasma virus production, resulted in a moderate positive correlation that did not reach statistical significance (0.8286, p value 0.06; data not shown).

In the treated animals, there was not a statistically significant correlation between the vDNA levels in lymphoid and mucosal tissues at necropsy and week 1 viremia (Figure S4 in File S1, 0.6 with p value 0.2). One animal in the treated group, GN19, had relatively high vDNA levels in lymphoid tissues compared to week 1 plasma viremia, suggesting it was an outlier. When this animal was removed from analysis, the other 5 animals reached statistical significance (Figure S4 in File S1, 0.9801 with p value of 0.001). The absence of a correlation may be due to the narrow range of the measured values and the relatively small number of animals studied. However, there was a trend towards statistical significance between lymphoid tissue vDNA levels at necropsy and AUC of plasma viremia over the entire 32 week period (0.9429 with p value 0.02).

### PBMC vDNA decay kinetics in untreated and ART-treated animals

**Figure 4:**
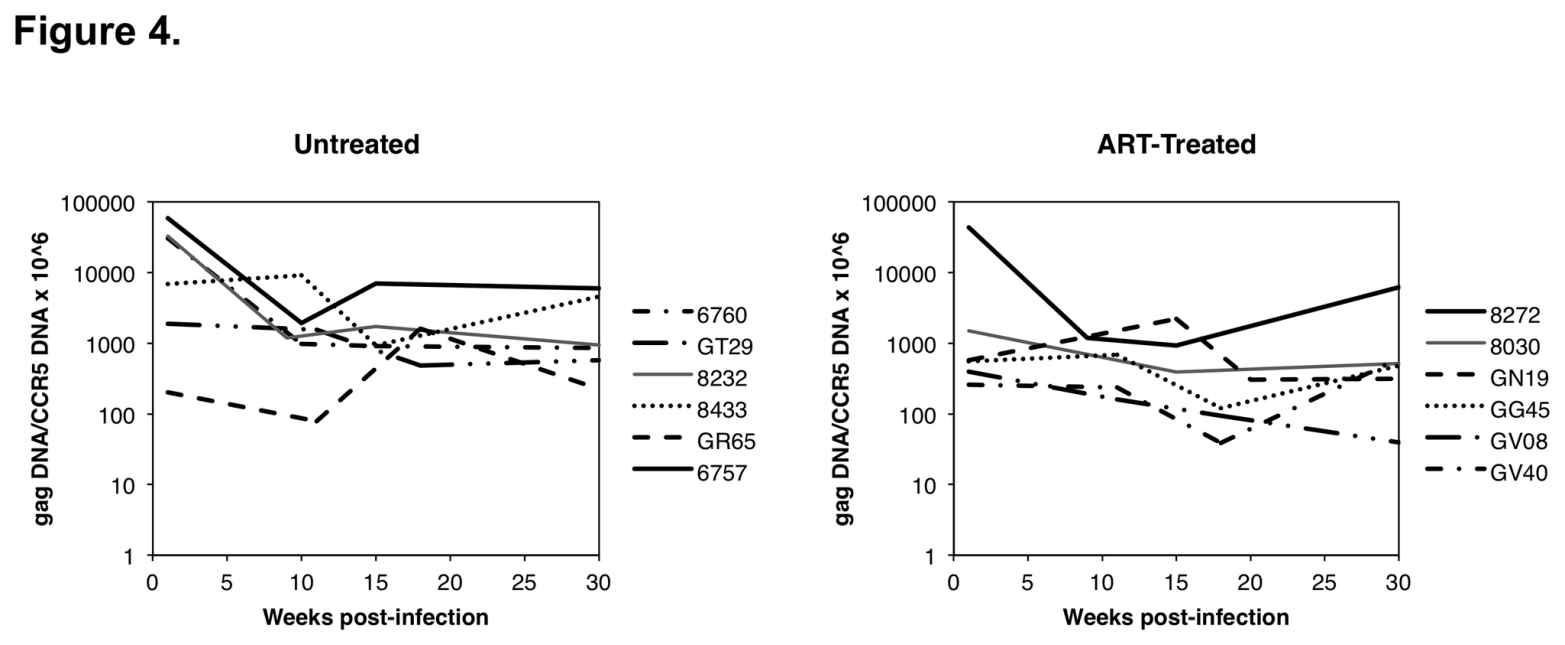
The ratio of RT-SHIV *gag* DNA copies per 10^6^ macaque CCR5 DNA copies were measured in PBMC isolated at different time points from each of the untreated and treated macaques.

vDNA levels in the PBMC of the untreated and ART-treated infected animals were measured at multiple time points (Figure 4). For the treated animals, PBMC were analyzed from 3 to 4 time points taken between weeks 1 and 30 post-infection, corresponding to early infection, to just prior to ART initiation, and to 17-20 weeks post-ART initiation. Fluctuations in PBMC vDNA levels were observed. Some animals from each group had little change in the PBMC vDNA levels, while others had up to a 1 log decline in infected cells over time. Only 4 of the animals (2 untreated, 2 treated) had detectable 2-LTR circles in the PBMC at 1 - 3 of the 4 tested time points, ranging from 70-700 copies per 10^6^ CCR5 copies (data not shown). Clearance of vDNA during the first 6 months of therapy of HIV+ individuals has been shown to be approximately 1 log and related to clearance of unintegrated and integrated HIV-1 genomes [33,34].

Reduced vDNA in PBMC did not appear to be a result of decreased CD4+ PBMC levels, as changes in PBMC vDNA levels did not reflect the multiple log reduction in plasma viremia observed in the treated animals and in some untreated animals that spontaneously controlled infection. This suggests that the number of infected PBMC in untreated animals is relatively stable or slightly declining over 30 weeks post-infection, similar to what is seen in HIV-1-infected individuals [35], and is not dramatically influenced by ART or correlated with changes in plasma vRNA levels during the treatment period. Levels of vDNA in PBMC were often lower than contemporaneous levels of vDNA in tissues (Table 1). This difference does not appear to be due to lower overall CD4+ lymphocyte percentages in the blood as compared to lymphoid tissues, as the PBMC CD4^+^ cell percentages of several animals were not vastly different from those observed in the LNs (1-2-fold lower; Figure 2C). Interestingly, the percentages of CD4+ T cells in the spleen were generally 2-3-fold lower than those of the lymph nodes. 

### Impact of ART on RT-SHIV replication, cellular expression and reservoirs

Tissues from untreated and treated macaques that had detectable vDNA were analyzed for gag RNA. To normalize for cell number, we measured *CD4* transcripts by qRT-PCR as an index of the frequency of potential RT-SHIV target cells. In general, at least 10^4^ *CD4* copies were detected in each tissue sample and the CD4 RNA levels in tissues correlated with the stable reference mRNA IPO-8 [36], suggesting that CD4 RNA expression was not selectively downregulated in some cells due to RT-SHIV infection (Table S3 in File S1). Normalized levels of gag RNA were determined for most tissues that had detectable vDNA for all treated and untreated animals (Table 2). In the untreated animals with detectable levels of plasma viremia at necropsy, vRNA was detected in multiple tissues in the gut, lung, and lymphoid tissues. Animals that were untreated but spontaneously suppressed virus replication to < 30 copies/mL plasma at necropsy had undetectable RNA in any tissues (6757) or had low levels (10-80 copies per 10^6^ CD4 copies) detectable only in spleen and a few lymph nodes (8433). 

**Table 2:** *gag* RNA copies per 1x10^6^ *CD4* RNA copies per tissue at necropsy (week 30/31).

	**Untreated**							**Treated**					
								**3 Drugs**		**4 Drugs**			
	**6760**	**8433**	**8232**	**6757**	**GT29**	**GR65**		**8272**	**8030**	**GN19**	**GG45**	**GV08**	**GV40**
Plasma RNA	**180000**	<30	**8200**	<30	**2900**	**50**		<30	<30	<30	<30	<30	<30
PBMC	**190**	na	**10**	-	-	-		-	**30**	-	-	-	-
Duodenum	**2040**	-	-	nd	nd	nd		-	nd	na	na	-	nd
Jejunum	na	-	**10**	nd	nd	nd		-	-	**10**	nd	nd	nd
Ileum	na	na	**270**	nd	-	nd		nd	nd	-	nd	-	nd
Colon	na	na	-	nd	**8200**	-		-	nd	**130**	nd	-	-
Cecum/Rectum	na	-	-	nd	**13000**	-		nd	-	nd	nd	-	-
Liver	-	-	nd	nd	-	nd		-	-	-	nd	nd	nd
Lung	**2990**	-	**1420**	-	-	-		-	-	**-**	-	-	nd
Thymus	**1370**	na	na	na	nd	nd		na	**10**	na	nd	nd	nd
Bone marrow	**180**	-	**10**	-	nd	nd		-	-	-	nd	nd	nd
Spleen	**5670**	**10**	**460**	-	nd	-		-	**10**	**190** ^a^	-	nd	-
Axillary LN	**15680**	**60**	**1200**	-	na	**170**		-	-	**30** ^a^	-	-	-
Bronchial LN	**19250**	**30**	**10**	-	nd	nd		-	-	-	nd	-	-
Inguinal LN	**399730**	-	**20**	-	**30** ^a^	**30**		-	-	na	-	na	**30**
Mandibular LN	**7400**	**80**	**110**	-	**120**	-		-	-	**130** ^a^	-	**30**	**10**
Mediastinal/Colon LN	na	-	nd	-	nd	-		-	-	-	-	-	**10**
Mesenteric LN	**23490**	-	**130**	-	**2390**	nd		-	-	-	-	-	-
Cerebellum	nd	nd	nd	nd	nd	nd		nd	nd	nd	-	nd	nd
Testicle	nd	nd	nd	nd	nd	nd		nd	-	nd	nd	nd	nd
Seminal vesicle	nd	nd	nd	nd	nd	nd		-	-	nd	nd	nd	nd

na, not available due to insufficient host RNA recovery or lack of tissuend, not determined due to low/no detectable vDNA

-, less than 1

^a^ less than 10^4^ CD4 RNA copies detected in the sample

The animals treated with 3 or 4 antiretroviral drugs had undetectable plasma viremia at the time of necropsy. The majority of tissues that were positive for vDNA from the ART-treated animals did not have detectable vRNA. Nevertheless, 4 of the 6 treated animals (one treated with 3 drugs, 3 treated with 4 drugs) had at least one lymphoid tissue that had detectable vRNA, spleen, thymus, or lymph node. Animal GN19, which among the treated animals had the highest peak viremia and set point prior to ART, also had detectable vRNA in the jejunum and colon. However, in general the levels of tissue vRNA in the treated animals were lower than those observed in the untreated animals. The ratio of vRNA to vDNA was determined for tissues taken from the animals at necropsy (Table S4 in File S1). Four of the 6 untreated animals had ratios of 1 or above for multiple tissues, whereas the 5 of 6 treated animals had no ratios above 1 in any tissue and one had a positive ratio in one lymph node. In comparing vRNA in the treated animals and the untreated animals (17 degrees of freedom) or in animals treated with 3 drugs vs. 4 drugs (10 degrees of freedom), the chi-square test results indicate that the distribution of RNA levels is highly variable among tissue type in both treated and untreated animals and is highly dependent on tissue type (p < 0.0005; Tables 1 and 2). Due to small counts in some tissues, Fisher’s exact test was also conducted, which was also significant (p < 0.0005). The variability was also observed in individual animals, in which most lymphoid tissues had higher number of infected cells and vRNA expression and the GI tract contained variable levels of infected cells and variable levels of vRNA expression. Examination of the data also indicated that some of the significance is likely due to differences in the among-tissue variation between treated and untreated samples. It appears that multiple tissues can serve as reservoirs capable of virus expression in both treated and untreated animals.

Replication-competent virus was isolated from resting CD4+ cells from lymph nodes in 5/5 untreated animals, including the two with low plasma viremia (8433 and 6757) and from 2/4 treated animals prior to ART initiation at week 12 or 13 (Table 3). In the two treated animals with detectable replication-competent virus (GN19 and GG45), the number of IUPM declined over time. None of the treated animals had detectable replication-competent virus culturable from LNs taken at necropsy, suggesting that very limited numbers of cells could be induced to produce replication-competent virus after multiple weeks of suppressive ART.

**Table 3:** Detection of replication-competent RT-SHIV from resting CD4+ T cells from lymph node biopsies.

		Week 12-13		Week 16-17		Week 30-32	
Treatment	Animal	Plasma vRNA	IUPM	Plasma vRNA	IUPM	Plasma vRNA	IUPM
	GT29	**660**	**420**	**810**	**421**	**2900**	**2500**
	8232	**90**	**2**	**370**	nd	**8200**	**8**
None	8433	**140**	**1.6**	**40**	nd	<30	**8**
	GR65	**40**	**0.5**	**100**	**3.2**	**50**	**16**
	6757	< 30	< 0.5	< 30	nd	<30	**8**
	GN19	**4500**	**206**	< 30	**2**	< 30	< 0.5
4 drugs	GG45	**60**	**8**	< 30	**3**	< 30	< 0.5
	GV08	< 30	< 0.5	< 30	< 0.5	< 30	< 0.5
	GV40	< 30	< 0.5	< 30	nd	< 30	< 0.5

nd, assay not done

### Viral RNA detection in PBMC did not correlate with treatment or plasma viremia

**Figure 5:**
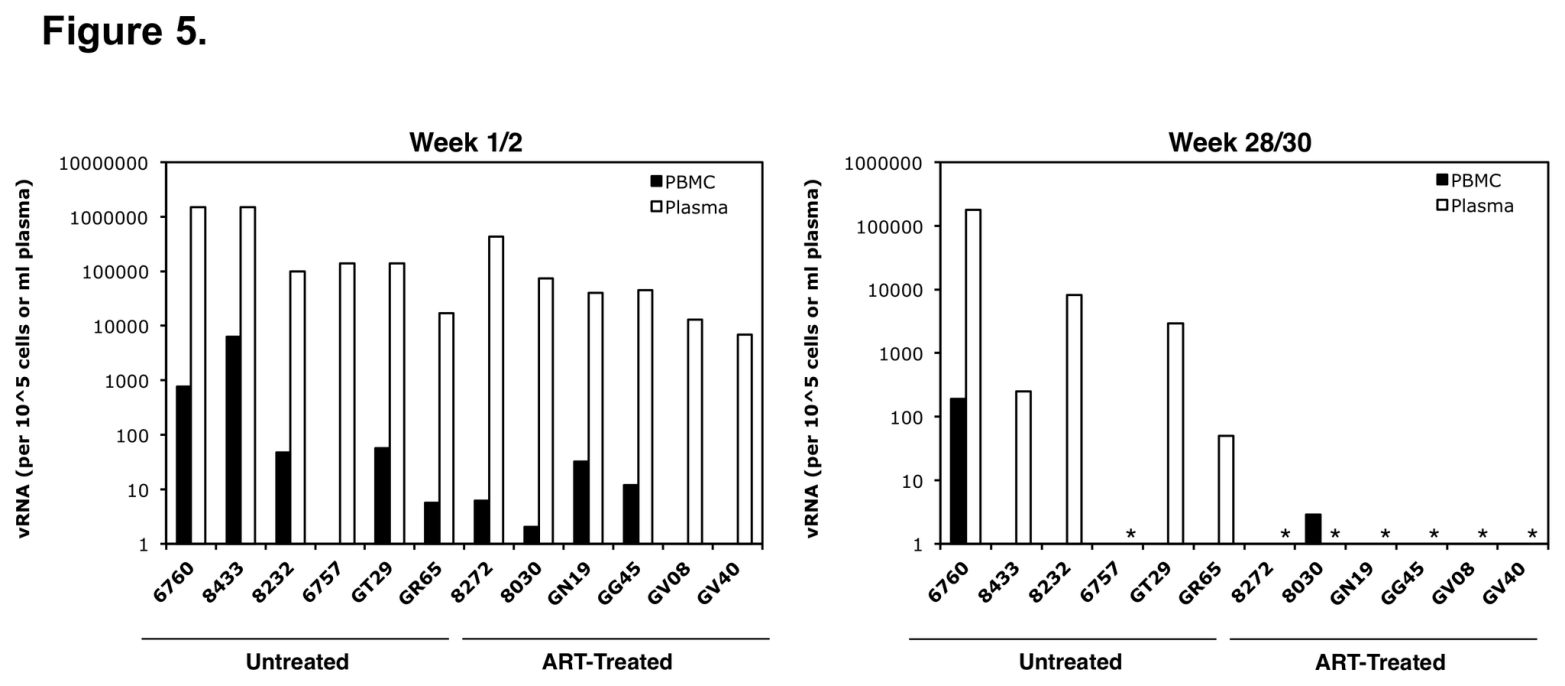
The ratio of RT-SHIV *gag* RNA copies per 10^6^ macaque CD4 RNA copies were measured in PBMC isolated at week 1 or 2 and 28 or 30 post-infection from each of the untreated and treated macaques (black bars). The amount of RT-SHIV gag RNA in the plasma is also plotted for each animal at each time point (white bars). Asterisks (*) denote plasma viremia levels below the limit of detection (<30 copies/ml).

While tissue vRNA levels generally reflected the level of plasma viremia at necropsy in the macaques, vRNA detection in PBMC varied greatly during early infection and at necropsy and did not seem to reflect plasma viremia (Figure 5). At week 1 or 2, plasma viremia ranged between 6.9 × 10^3^ to 1.5 × 10^6^ vRNA copies per ml in the animals, whereas vRNA in the PBMC ranged from 2 × 10° to 6.3 × 10^5^ copies per 10^5^ *CD4* copies and did not correlate with the level of plasma viremia. The wide variation of PBMC vRNA (> 5 logs) was reproducible in multiple aliquots tested (data not shown) and was not due to variation in CD4+ T levels in the blood, as the gag RNA levels were normalized per cellular CD4 RNA level. Also, the absolute CD4+ T cell count in the PBMC of the animals ranged from 816 to 3877, which is less than a 5-fold difference (Figures 2A and 2B). The lack of correlation between vRNA detected in the plasma and in the PBMC was even greater at week 28-31 post-infection (Figure 4B). Of the 5 untreated animals that had measurable plasma viremia (> 30 copies/ml), only 2 animals had detectable PBMC vRNA (> 1 copy/10^5 CD4 copies). And of the 7 animals that had no detectable plasma vRNA (6 treated and 1 untreated), one had detectable vRNA in the PBMC. The PBMC vRNA levels also did not reflect the amount of vRNA or vDNA detected in the tissues (Tables 2 and 3). 

## Discussion

The nature and size of persisting cellular and tissue compartments that despite apparently effective ART harbor persistent infected cells and that can give rise to recrudescent viremia when treatment is stopped (i.e. viral reservoirs) need to be determined to successfully target them for eradication. Measurement of vDNA+ cells provides a convenient, albeit imperfect, index of the frequency of such cells. However, detection of vDNA+ cells in the blood may not reflect replication elsewhere in the body [37], particularly if tissue compartmentalization of virus replication occurs [38-42] or drug penetration into tissues is not optimal [43]. Because tissue sampling from multiple organs in humans is difficult to perform, animal models for HIV/AIDS, in which more extensive tissue sampling is feasible, may usefully inform studies of viral persistence on suppressive ART, and evaluation of strategies to target this residual virus. Similarities in anatomy, immune responses, and key features of pathogenesis makes nonhuman primate models ideal for identifying reservoirs and assessing the efficacy of novel strategies to reduce them.

To characterize infected cells in RT-SHIV_mne_-infected macaques in the absence of any antiretroviral treatment or during suppressive ART, we examined multiple tissues for the presence of RT-SHIV DNA and RNA after no treatment or 17-20 weeks of suppressive therapy with 3 or 4 antiviral drugs. As in available surveys of PBMC and tissues from HIV-infected individuals [44,45], there was substantial inter-animal variation in vDNA levels. PBMC, lymphoid tissues (mainly spleen and lymph nodes) and the gastrointestinal tract contained the highest levels of vDNA in untreated as well as in treated macaques. Lung tissue, bone marrow, and brain tissue of some of these animals showed detectable vDNA but at a much lower level than the other tissues mentioned above. Little or no RT-SHIV_mne_ DNA was detected in brain, kidney, or male genital tract tissues, which may be due to fewer target CD4+ cells in these organs or in the specimens sampled from these organs. 

These vDNA data are consistent with findings from other macaque studies. In a study of rhesus macaques infected with RT-SHIV_mac239_ and treated with FTC, TFV and EFV, lymphoid and gastrointestinal tissues showed the highest level of vDNA in virally suppressed animals [46]. Also, in a SIV model with FTC and tenofovir therapy, it was shown that resting CD4+ T lymphocytes from lymph nodes but not thymocytes contribute to reservoirs [27]. We previously observed vDNA and vRNA in multiple lymphoid tissues and in the gastrointestinal tract, and we have seen intestinal pathology with high RT-SHIV viremia [24]. In a model of neuropathogenic AIDS, although neurotropic virus in plasma and cerebral spinal fluid was significantly reduced by therapy (TFV, saquinavir, atazanavir and an integrase inhibitor), levels of viral DNA in the brains of these animals were not significantly different than the untreated controls, suggesting that virally infected cells in the central nervous system represent a potential reservoir [47]. In the current study, we did not see RT-SHIV_mne_+ cells in the brain, likely due to a lack of neurotropism of this virus as evidenced by lower levels of vRNA in the cerebral spinal fluid of animals infected with our virus compared to other strains (Ambrose and Lifson, unpublished results). While the RT-SHIV_mne_ model appears relevant for studying reservoirs, particularly in animals with high viremia and complete ART suppression, as with HIV-1, not all SIVs or SHIVs, including RT-SHIV_mne_, consistently cause CNS disease [48,49]. Studies with different viruses that provide consistent neurological disease should be employed for focusing studies on CNS reservoirs [50].

The level of vDNA in lymphoid tissues was correlated with the level of week 1 plasma viremia, suggesting that viral reservoirs are established early and may be determined by the level of early virus replication as reflected by the plasma viremia. In a different nonhuman primate model, Bourry et al. also showed that tissue reservoirs are established within 1 week post-infection [51]. They treated cynomolgus macaques with ART (zidovudine, lamivudine and indinavir) beginning at 14 days after infection with SIV_mac251_ and observed no significant differences in vDNA levels in the spleen, peripheral and mesenteric LN, ileum, and PBMC of treated vs. untreated animals, suggesting that establishment of reservoirs had already occurred. When treatment was initiated at 1 week post-infection, there were only modest or no differences between the tissue levels of vDNA measured for the two groups. This finding is consistent with our observed correlation between early plasma viremia and level of vDNA in tissue reservoirs. 

While we observed consistent and high levels of vDNA in lymphoid tissues, there was a high degree of variability among GI tract tissues, in which many specimens were negative for infected cells. It is unlikely that significant loss of CD4+ cells resulted in this observation, as CD4+ T cells were still relatively abundant in the infected animals. This could be due to an uneven distribution of CD4+ target cells and infected foci throughout the GI tract, coupled with random sampling. Alternatively, gut tissues may not contribute as significantly as lymphoid organs to the reservoirs established by RT-SHIV_mne_. 

Tissue RT-SHIV_mne_ vRNA levels were lower in ART-treated macaques than in untreated macaques. However, 6 treated animals had detectable vRNA in some lymphoid tissues and 2 of these also had detectable vRNA in mucosal tissues, albeit at lower levels than the untreated animals. This general effect of treatment is consistent with a prior rhesus RT-SHIV_mac239_ study, in which lower but detectable levels of vRNA were observed in many of the tissues of ART-treated animals compared to tissues of an untreated animal [46], and a suggestion that penetration of antiretroviral drugs into different tissues may be variable. In that study, the treated rhesus macaques had significant levels of plasma viremia during the first 10 weeks of ART, likely due to incompletely effective treatment of high levels of viral replication, reflected in extremely high plasma viral loads in contrast to this study. It is unclear if observed residual vRNA in our study is due to spontaneous reactivation of virally infected cells or if there is ongoing local replication in tissues. Previously we demonstrated that little or no evolution of plasma viral sequences occurs in RT-SHIV-infected macaques receiving ART that are suppressed for up to 20 weeks [23]. Recently, two clinical studies showed little evolution of viral sequences obtained from gut biopsies collected longitudinally from HIV-infected individuals, a finding interpreted as indicating that ongoing replication does not occur in the gastrointestinal tract [52,53]. Further studies will need to be performed on sequences or replication competent-virus isolated from tissues of RT-SHIV-infected macaques to determine if ongoing replication is occurring or not.

The number of animals studied did not provide statistical power for a rigorous comparison between the 3 and 4 drug regimens. However, effective suppression was achieved in both groups of animals and it seems unlikely in this study with relatively low viremia that intensification with a fourth drug significantly affected residual plasma viremia, as many HIV-1 clinical studies have shown previously [14,54-57]. Interestingly, one study suggested that ongoing replication could be occurring during suppressive therapy in the gastrointestinal tract during ART or reflected in increases in PBMC 2-LTR circles, which could be decreased during intensification with an integrase inhibitor [10,14,58]. Future studies to address tissue replication or decrease of reservoirs with treatment intensification in macaques, including correlation with tissue drug levels, will be needed to address this issue further.

Surprisingly, the PBMC vDNA decay kinetics was similar in the untreated group and the ART-treated group between weeks 1 and 30. While the levels fluctuated greatly over time, some animals showed little decay and some animals showed significant decay. However, the decrease in circulating virally infected cells did not correlate with the concomitant multiple log decrease in plasma viremia that occurred in many of the animals. Levels of PBMC vDNA at necropsy did not correlate with tissue vDNA levels, plasma vRNA levels, or recovery of replication-competent virus from resting CD4+ cells in LN. These results are consistent with studies that show that vDNA and vRNA levels are significantly higher in gut tissues than those in PBMC in HIV-infected individuals on suppressive ART [59,60]. In addition, some untreated animals with relatively high plasma viremia had little to no detectable vRNA in the PBMC taken at the same time points. These results suggest that the PBMC are not reflective of viral tissue reservoirs and that productively infected PBMC contribute little to the overall plasma virus pool. A previous study demonstrated that cellular reservoirs in ART-treated HIV+ individuals are heterogeneous and have differential decay kinetics [61]. Additional studies to compare the RT-SHIV RNA and DNA sequences from different anatomical compartments as well as the blood are needed to address what reservoir(s) contribute to virus-infected PBMC. In addition, other experiments are needed to determine if the length of time of viral latency affects the ability of provirus to be reactivated in vitro or in vivo.

One possible confounder of the vRNA and vDNA results in this study and previous studies is that the animals were not perfused during necropsy to remove residual blood from inside vessels within tissues. Phylogenetic analysis of single-genome sequences from both treated and untreated animals in this study were performed (Kearney et al., in preparation). To determine the effect of possible contaminating blood cells on the sequences obtained from tissues, varying numbers of sequences from blood and tissues were compared hundreds of times. No differences were found between populations even at very small numbers, which likely eliminated the possible contaminants, indicating that any sequences resulting from PBMC contamination in the tissues does not strongly influence the power of these studies to detect a difference between the populations. Very little viral RNA was detected in most tissues of ART-treated animals and some tissues of untreated animals, suggesting little contamination of tissues with blood. Furthermore, viral RNA sequences recovered from the tissues of treated animals show specific defects (e.g. deletions, G to A hypermutations) that were not detected in blood, implying that virus detected in tissue is not due to blood contamination.

Macaque models, such as RT-SHIV_mne_ infection of pigtailed macaques described here, are useful for addressing questions concerning viral reservoirs, particularly in providing sampling for numerous tissues that are not readily or adequately sampled in a clinical setting. The limited number of animals and variability in viremia levels impact the interpretation of the study. As host genetic factors can influence levels of viral infection in macaques [62], variability of RT-SHIV viremia may be due to undefined host genetic factors in pigtailed macaques. While many previous studies looking at tissue reservoirs with other macaque models have not demonstrated complete suppression during ART nor the use of an IN inhibitor with commonly prescribed triple combination therapy, the level of virus suppression achieved with clinically relevant ART in the present study sets the stage for defining the different cellular compartments that comprise viral reservoirs in ART-suppressed individuals. We have determined that persisting viral reservoirs consist of multiple lymphoid and mucosal tissues containing vDNA but little or no detectable vRNA and that few infected cells are likely contributing to viremia in the presence or absence of an IN inhibitor. With variable plasma viremia levels observed in our macaque model, as are exhibited in HIV-infected individuals, our data also suggest that greater replication of virus early after infection influences the size of the viral reservoirs. Ongoing and future studies with complete viral suppression in infected macaques will be critical to defining the cellular reservoirs, determining whether these infected cells are different in various tissues vs. the blood, whether early initiation of ART can reduce reservoirs, what therapies can be used to selectively eradicate these cells, and whether infected cells that are not actively producing virus are able to be stimulated and eliminated.

## Supporting Information

File S1 **Figure S1** , (A) Host CCR5 and RT-SHIVmne gag DNA were quantified in each tissue obtained from animal 6760. Each bar represents the average of duplicates and error bars represent the standard deviations. The limit of quantification for (B) CCR5 was 10 copies and for (C) *gag* was 1 copy. **Figure** **S2**, (A) RT-SHIVmne *gag* and 2-LTR circle copies were measured in tissues from animal 6760. Each bar represents the average of duplicates and error bars represent the standard deviations. (B) The limit of quantification of the 2-LTR circle assay was 1 copy. **Figure** **S3**, (A) Host CD4 and RT-SHIVmne gag RNA levels were quantified in each tissue obtained from animal 6760. Each bar represents the average of duplicates and error bars represent the standard deviations. The limit of quantification for (B) CD4 was 10 copies and for (C) *gag* was 1 copy. **Figure** **S4**, (A) The ratio of *gag* copies per 106 CCR5 copies for each tissue of the ART treated RT-SHIV-infected macaques. The average of each qPCR reaction was used for the graph. In addition, the week 1 plasma viral load was included for each animal. Asterisks (*) denote samples that were not collected or in which no significant CCR5 DNA were measured. (B) The amount of *gag* vDNA detected in each of the lymphoid tissues for each animal was plotted against the week 1 plasma viremia level for all animals (left panel) or excluding GN19 (right panel), giving a Spearman rank-order correlation of 0.6 with a p value of 0.2. (C) The amount of *gag* vDNA detected in each of the lymphoid tissues for each animal was plotted against the area under the curve (AUC) of plasma viremia between weeks 1-32 postinfection, giving a Spearman rank-order correlation 0.771 with a p value of 0.07.
(PDF)
