# Supplementary material for: Persistence of Viral Reservoirs in Multiple Tissues after Antiretroviral Therapy Suppression in a Macaque RT-SHIV Model
Source: PLoS One. 2013 Dec 18;8(12):e84275. doi: 10.1371/journal.pone.0084275 (PMC3867492; doi:10.1371/journal.pone.0084275)
Supplement: File S1 — Figure S1 , (A) Host CCR5 and RT-SHIVmne gag DNA were quantified in each tissue obtained from animal 6760. Each bar represents the average of duplicates and error bars represent the standard deviations. The limit of quantification for (B) CCR5 was 10 copies and for (C) gag was 1 copy. Figure S2, (A) RT-SHIVmne gag and 2-LTR circle copies were measured in tissues from animal 6760. Each bar represents the average of duplicates and error bars represent the standard deviations. (B) The limit of quantification of the 2-LTR circle assay was 1 copy. Figure S3, (A) Host CD4 and RT-SHIVmne gag RNA levels were quantified in each tissue obtained from animal 6760. Each bar represents the average of duplicates and error bars represent the standard deviations. The limit of quantification for (B) CD4 was 10 copies and for (C) gag was 1 copy. Figure S4, (A) The ratio of gag copies per 106 CCR5 copies for each tissue of the ART treated RT-SHIV-infected macaques. The average of each qPCR reaction was used for the graph. In addition, the week 1 plasma viral load was included for each animal. Asterisks (*) denote samples that were not collected or in which no significant CCR5 DNA were measured. (B) The amount of gag vDNA detected in each of the lymphoid tissues for each animal was plotted against the week 1 plasma viremia level for all animals (left panel) or excluding GN19 (right panel), giving a Spearman rank-order correlation of 0.6 with a p value of 0.2. (C) The amount of gag vDNA detected in each of the lymphoid tissues for each animal was plotted against the area under the curve (AUC) of plasma viremia between weeks 1-32 postinfection, giving a Spearman rank-order correlation 0.771 with a p value of 0.07. (PDF) [file pone.0084275.s001.pdf]

Table S1. Primers and probes used for qRT-PCR and qPCR.

Coding region	Primer/probe	Sequence
Macaque CD4	forward	5'-ACATCGTGGTGCTAGCTTTCCAGA-3'
	probe	5'F-AGGCCTCCAGCACAGTCTATAAGAAAGAGG-T3'
	reverse	5'-AAGTGTAAAGGCGAGTGGGAAGGA-3'
Macaque IPO-8	forward	5'-GCTCTGATAACTGTGCAG-3'
	probe	5'F-TGCTCTCCTCTGATCCTCGC-T3'
	reverse	5'-CAGTGTGTACACCTCCTG-3'
RT-SHIV$_{mne}$ Gag	forward	5'-GTCTGCGTCATCTGGATTC-3'
	probe	5'F-CTTCCTCAGTGTGTTTCACTTTCTCTTCTGCG-T3'
	reverse	5'-CACTAGGTGTCTCTGCACTATCTGTTTTG-3'
Macaque CCR5	forward	5'-ATGGACTATCAAGTGTCAAGTC-3'
	probe	5'F-CGCCTCCTGCCTCCGCTCTA-T3'
	reverse	5'-TCACAAGCCCACAGATATTTC-3'
RT-SHIV$_{mne}$ 2-LTR circle junction	forward	5'-CGCTGGCTTGCTTAAAGACCTC-3'
	probe	5'F-AAGCTGCCTTTTAGAAGTAAGCCAGTGTGTGC-T3'
	reverse	5'-GATACATTTACAGGGACTAATTTCCATAGC-3'

F, fluorescein (FAM); T, rhodamine (TAMRA)

Table S2. qPCR on replicates of viral DNA and RNA standard dilutions.

Assay	No. of replicates positive for 1 copy/well	No. of replicates positive for 10 - 10^6 copies/well
gag DNA	6/10	10/10
2-LTR DNA	8/10	10/10
gag RNA	7/10	10/10

Table S3. IPO-8 RNA to CD4 RNA ratio in macaque tissues.

RNA Sample	Average CD4 threshold cycle (C_t) ± stdev[a]	Average IPO-8 threshold cycle (C_t) ± stdev[a]	Average IPO-8 C_t/ Average CD4 C_t
1	29.00 ± 0.07	28.96 ± 0.20	1.00
1 repeat[b]	ND	29.53 ± 0.50	1.02
2	31.75 ± 0.13	38.82 ± 0.38	1.22
3	28.20 ± 0.11	31.46 ± 0.38	1.12
4	29.73 ± 0.16	38.04 ± 0.15	1.28
5	29.27 ± 0.00	29.47 ± 0.15	1.01
6	29.76 ± 0.12	29.49 ± 0.06	0.99
Average	-	-	1.09

[a] Average of duplicate wells measured by qRT-PCR

[b] Second IPO-8 qRT-PCR of RNA sample compared to same CD4 qRT-PCR

ND, not done

Table S4. *gag* RNA to *gag* DNA copy ratio per tissue at necropsy (week 30/31)

	**Untreated**						**Treated**					
							**3 Drugs**		**4 Drugs**			
	**6760**	**8433**	**8232**	**6757**	**GT29**	**GR65**	**8272**	**8030**	**GN19**	**GG45**	**GV08**	**GV40**
Plasma RNA	**180000**	<30	**8200**	<30	**2900**	**50**	<30	<30	<30	<30	<30	<30
PBMC	0.7	na	0.02	-	-	-	-	0.6	-	-	-	-
Duodenum	**32**	-	-	*	-	-	-	-	nd	nd	-	-
Jejunum	na	-	**1**	-	-	nd	-	-	0.02	-	-	-
Ileum	na	na	**1**	*	-	-	na	-	-	nd	-	-
Colon	na	na	-	*	**513**	*	-	-	0.02	-	-	-
Cecum/Rectum	na	-	-	nd	**2600**	-	na	-	nd	-	-	-
Liver	-	-	nd	-	-	*	-	-	-	nd	-	-
Lung	**332**	-	**37**	-	-	nd	-	-	-	-	-	-
Thymus	**196**	na	na	na	-	-	na	0.05	na	-	-	-
Bone marrow	**7**	-	**5**	-	-	*	-	-	-	-	-	-
Spleen	**21**	0.05	**7**	-	-	-	-	0.2	0.002	-	-	-
Axillary LN	**19**	0.2	**14**	-	na	**34**	-	-	0.009	-	-	-
Bronchial LN	**19**	0.03	0.1	-	-	-	-	-	-	-	-	-
Inguinal LN	**654**	-	**1**	-	0.8	**3**	-	-	na	-	-	**5**
Mandibular LN	**8**	0.1	**1**	-	**7**	-	nd	-	0.3	-	-	0.4
Mediastinal/ Colon LN	na	-	na	-	-	-	nd	-	-	-	-	0.3
Mesenteric LN	**44**	-	0.5	-	**598**	-	-	-	-	-	-	-

na, not available due to insufficient vRNA or vDNA recovery or lack of tissue
nd, not determined
*, ≤ 2 copies of vDNA detected

Supplemental Figure 1.

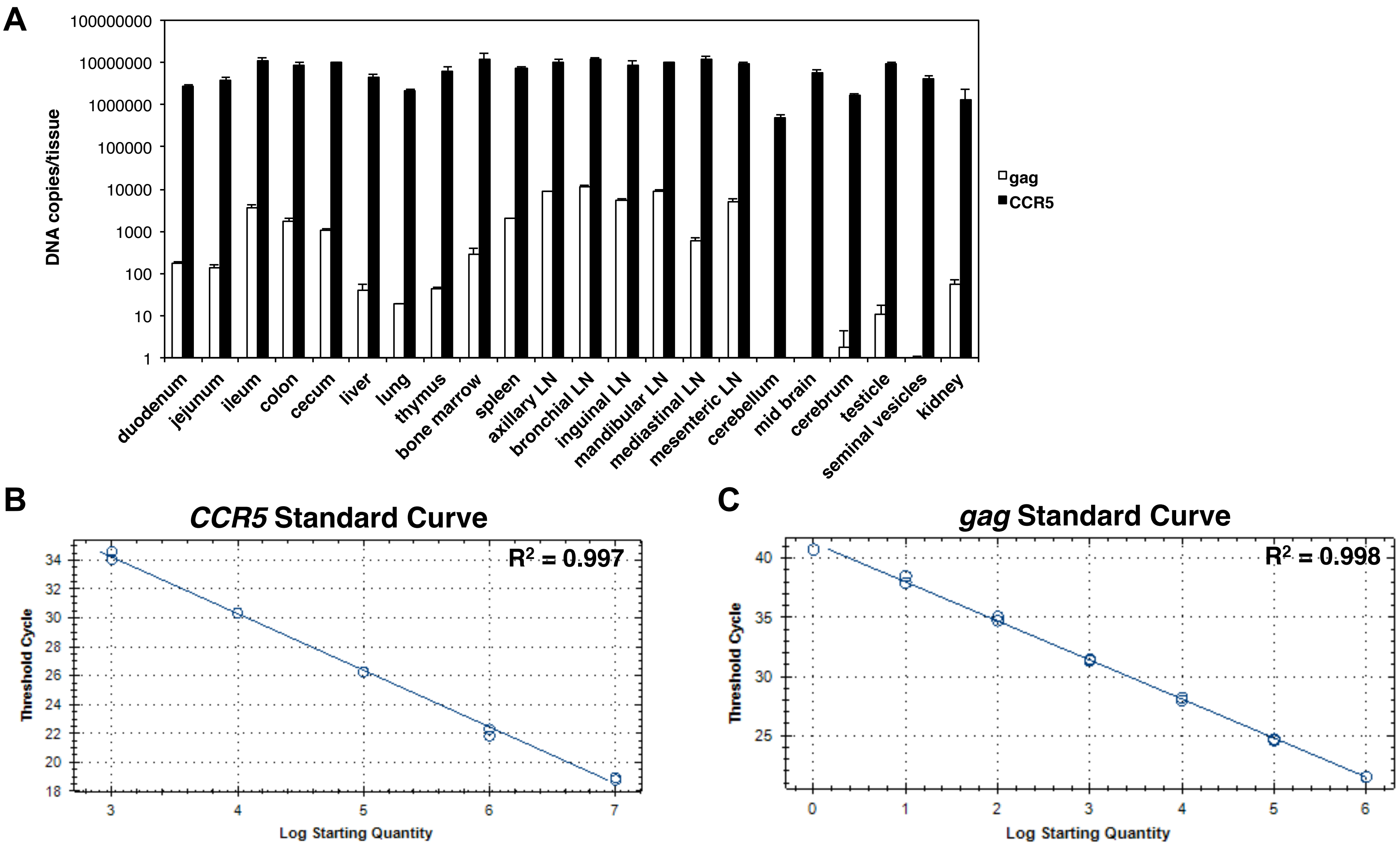

Figure S1. (A) Host *CCR5* and RT-SHIV$_{mne}$ *gag* DNA were quantified in each tissue obtained from animal 6760. Each bar represents the average of duplicates and error bars represent the standard deviations. The limit of quantification for (B) *CCR5* was 10 copies and for (C) *gag* was 1 copy.

Supplemental Figure 2.

A

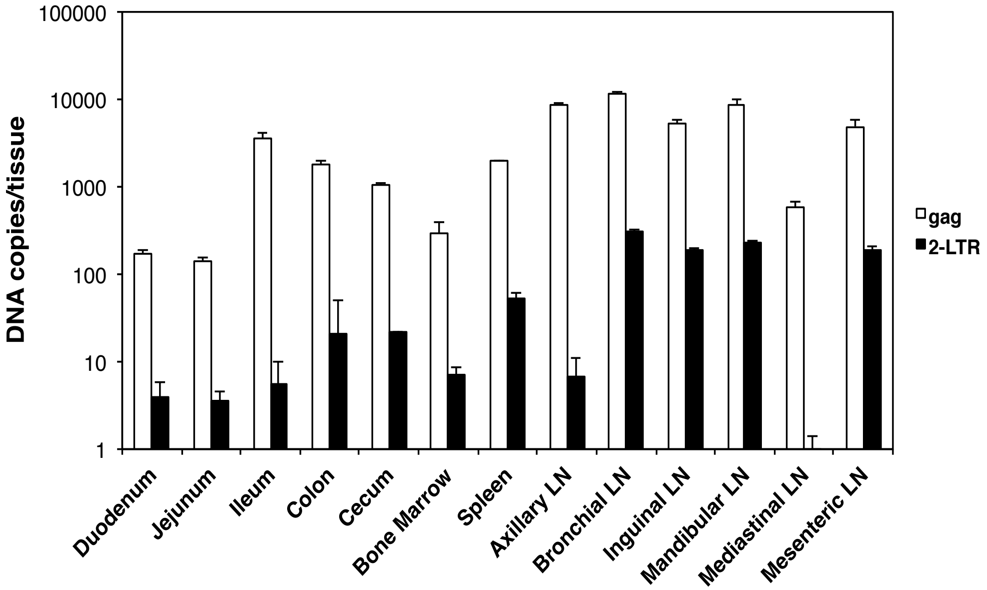

B

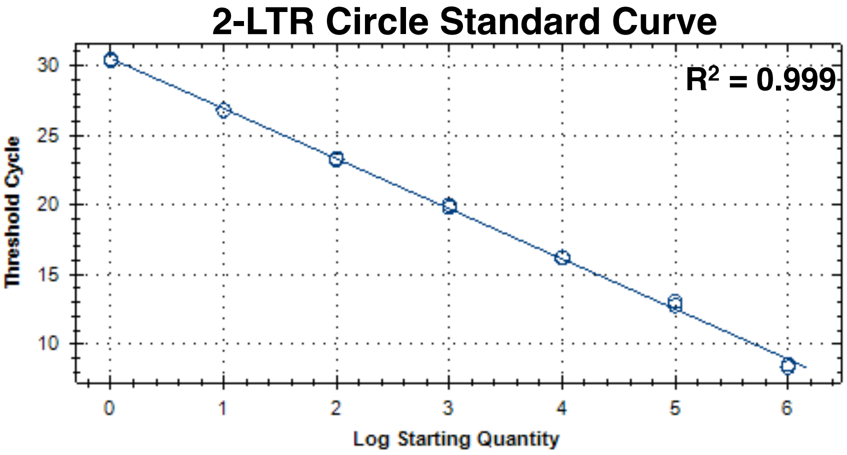

Figure S2. (A) RT-SHIV$_{mne}$ *gag* and 2-LTR circle copies were measured in tissues from animal 6760. Each bar represents the average of duplicates and error bars represent the standard deviations. (B) The limit of quantification of the 2-LTR circle assay was 1 copy.

Supplemental Figure 3.

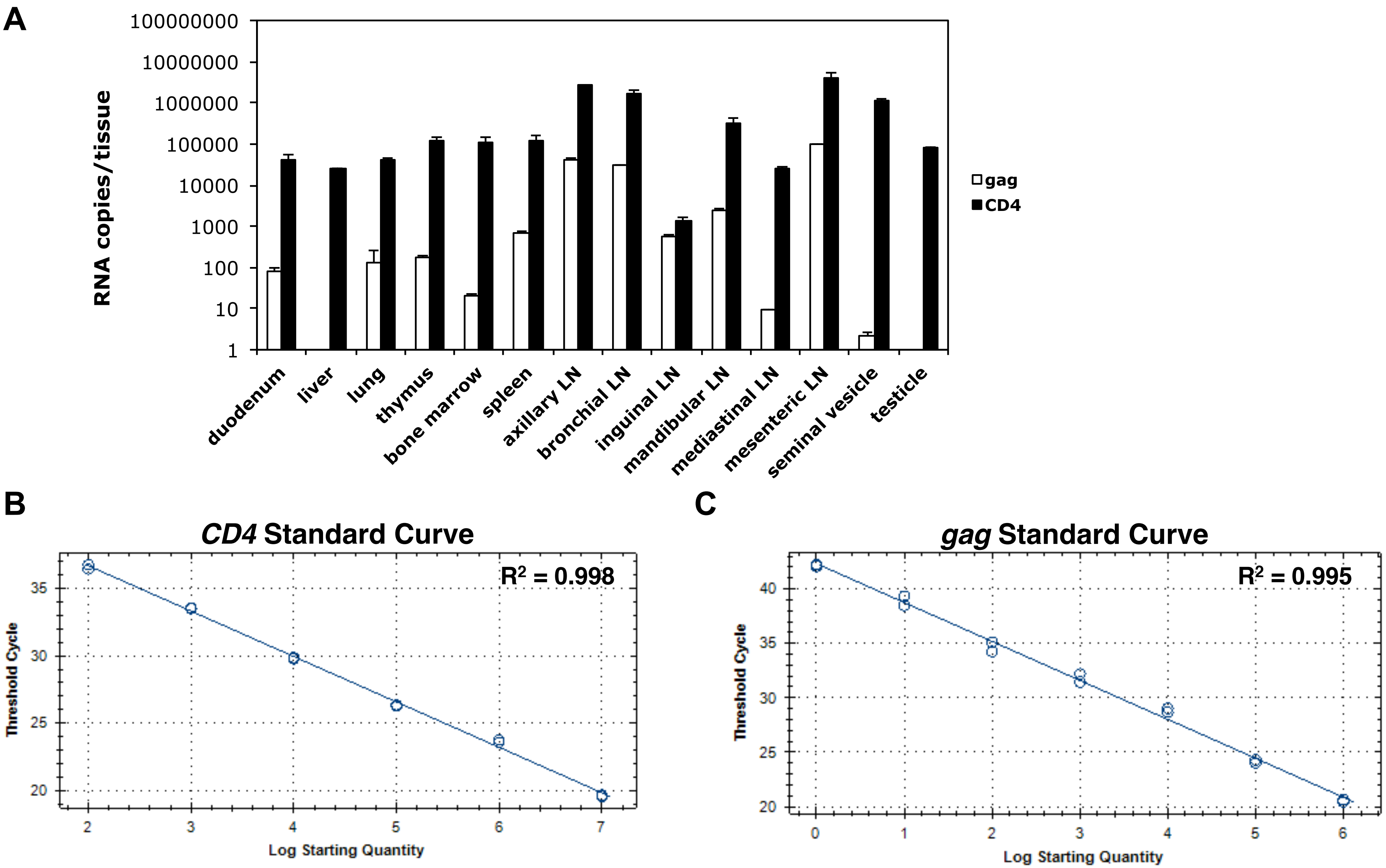

Figure S3. (A) Host *CD4* and RT-SHIV$_{mne}$ *gag* RNA levels were quantified in each tissue obtained from animal 6760. Each bar represents the average of duplicates and error bars represent the standard deviations. The limit of quantification for (B) *CD4* was 10 copies and for (C) *gag* was 1 copy.

Supplemental Figure 4.

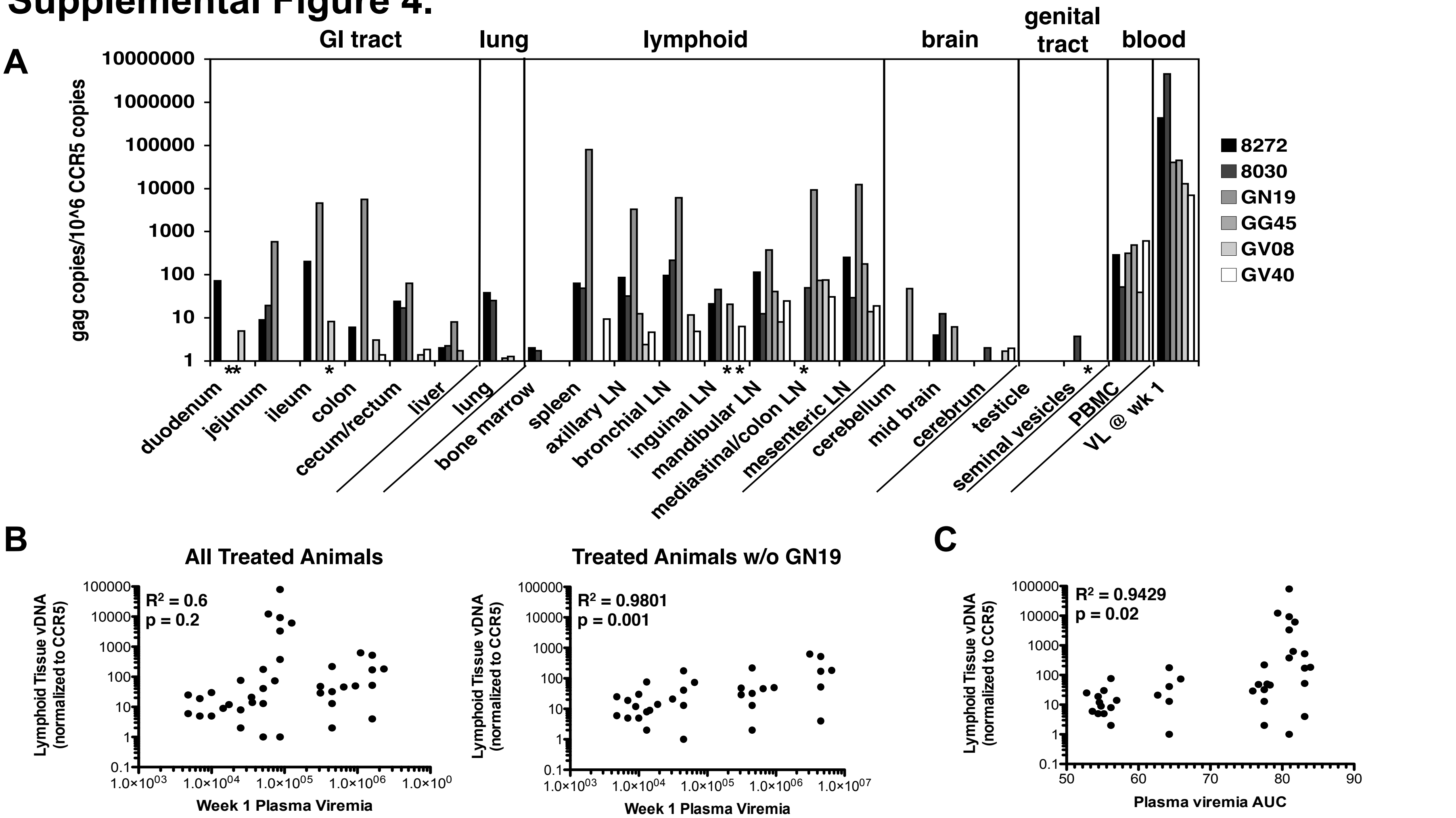

Figure S4. (A) The ratio of *gag* copies per 10^6 *CCR5* copies for each tissue of the ART treated RT-SHIV-infected macaques. The average of each qPCR reaction was used for the graph. In addition, the week 1 plasma viral load was included for each animal. Asterisks (*) denote samples that were not collected or in which no significant *CCR5* DNA were measured. (B) The amount of gag vDNA detected in each of the lymphoid tissues for each animal was plotted against the week 1 plasma viremia level for all animals (left panel) or excluding GN19 (right panel), giving a Spearman rank-order correlation of 0.6 with a p value of 0.2. (C) The amount of gag vDNA detected in each of the lymphoid tissues for each animal was plotted against the area under the curve (AUC) of plasma viremia between weeks 1-32 post-infection, giving a Spearman rank-order correlation 0.771 with a p value of 0.07.
